# Editorial: Integrative Computational Systems Biology Approaches in Immunology and Medicine

**DOI:** 10.3389/fmicb.2018.03338

**Published:** 2019-01-23

**Authors:** Lars Kaderali, Fabian Theis, Vitaly V. Ganusov, Stanca M. Ciupe, Ramit Mehr, Ruy M. Ribeiro, Esteban A. Hernandez-Vargas

**Affiliations:** ^1^Institute of Bioinformatics, Universitätsmedizin Greifswald, Greifswald, Germany; ^2^Helmholtz Zentrum München, Neuherberg, Germany; ^3^Department of Microbiology, University of Tennessee, Knoxville, Knoxville, TN, United States; ^4^Department of Mathematics, Virginia Tech, Blacksburg, VA, United States; ^5^The Mina & Everard Goodman Faculty of Life Sciences, Bar-Ilan University, Ramat Gan, Israel; ^6^Faculdade de Medicina da Universidade de Lisboa, Lisbon, Portugal; ^7^Frankfurt Institute for Advanced Studies, Frankfurt am Main, Germany

**Keywords:** mathematical modeling, systems biology, immunology, infectious diseases, virology

New technologies provide the ability to generate massive amounts of immunological data in health and disease. However, this “Big data” trend is becoming more challenging and unfeasible by the steep increase in the number of different pieces of information and the complexity of large datasets. Thus, systematic and tractable approaches that integrate a variety of biological and medical research data at multiple scales into mathematical, statistical, and computational models are crucial to harness knowledge and to develop new therapies.

A “Frontiers in Immunology” and “Frontiers in Microbiology” research topic was proposed to address the current state of the art of bioinformatics and computational models covering processes at multiple temporal and/or spatial scales (e.g., genes, molecular, cells, tissues, organs, individual, and population) and in combination with animal experiments and clinical data. An additional reason to prepare this research topic was to celebrate the 70th birthday of Alan Perelson, one of the pioneers of mathematical modeling in virology and immunology.

A total of 23 papers were accepted for publication, which attests to the timeliness of the topic. The papers included in this Research Topic reflect many of the open issues in theoretical immunology and infectious diseases. Some of the papers address challenging questions—such as the understanding of HIV and the immune system (Vaidya et al.;  Móréh et al.; Hernandez-Vargas; Yang and Ganusov; Cangelosi et al.; Ciupe et al.), the effect of parasites during a malaria infection (Thakre et al.), how immune cells operate in germinal centers (Amitai et al.; Joslyn et al.) or in the lymph nodes (Tasnim et al.); and the quantification of influenza viral dynamics (Smith et al.). Four papers proposed a multiscale modeling approach with the main goal to bring together data from different scales (Carruthers et al.; Quintela et al.; Lehnert and Figge; Zitzmann and Kaderali). The problem of traditional fitting methods for ODEs applied to noisy problems was discussed in this research topic (Romero-Severson et al.). Ultimately, numerical simulations (Pigozzo et al.; Timme et al.; Prauße et al.) and bioinformatic tools (Pandey et al.; Naz et al.; Farías et al.; Garg et al.) show the importance of systems biology in understanding experimental outcomes.

These papers provide a broad overview of current issues in systems biology and we would like to thank the Frontiers editorial staff, all the authors who contributed excellent papers, and the reviewers whose work has made publication of this research topic possible.

## Author Contributions

All authors listed have made a substantial, direct and intellectual contribution to the work, and approved it for publication.

### Conflict of Interest Statement

The authors declare that the research was conducted in the absence of any commercial or financial relationships that could be construed as a potential conflict of interest.

